# Comparative Molecular Insights and Computational Modeling of Multiple Myeloma and Osteosarcoma

**DOI:** 10.3390/ijms27083611

**Published:** 2026-04-18

**Authors:** Alina Ioana Ghiță, Vadim V. Silberschmidt, Mariana Ioniță

**Affiliations:** 1 Faculty of Medical Engineering, National University of Science and Technology Politehnica Bucharest, Gheorghe Polizu 1-7, 011061 Bucharest, Romania; alina_ioana.ghita@upb.ro (A.I.G.); v.silberschmidt@lboro.ac.uk (V.V.S.); 2 Faculty of Veterinary Medicine, Spiru Haret University, Basarabia 256, 031121 Bucharest, Romania; 3 Advanced Polymer Materials Group, National University of Science and Technology Politehnica Bucharest, Gheorghe Polizu 1-7, 011061 Bucharest, Romania; 4 Ebio-Hub Research Center, National University of Science and Technology Politehnica Bucharest-Campus, Iuliu Maniu 6, 061344 Bucharest, Romania

**Keywords:** bone, malignancy, multiple myeloma, osteosarcoma, in silico, models, AI

## Abstract

Multiple myeloma (MM) and osteosarcoma (OS) are two biologically distinct osseous malignancies with similar molecular networks that present translational challenges for their computational modeling. This comparative research analyzes MM and OS biology relevant to in silico approaches, focusing on PI3K-AKT-mTOR signaling, the RANK-RANKL-OPG axis, angiogenic factors (VEGF, TGFs), and immune mediators in MM, alongside the transcription factors (SOX9, RUNX2), signaling pathways (PI3K-AKT-mTOR, NOTCH), immune cell state (TAM2), and interleukins in OS. Based on this pathophysiologic foundation, the review outlines five computational paradigms: (i) mechanistic models; (ii) data-driven/machine learning schemes; (iii) hybrid mechanistic approaches; (iv) digital twins/virtual cohorts, and (v) MIDD/PBPK models for real-world applications. A cross-cancer comparison section summarizes common and distinct biological axes and their computational translation as well as the overlapping features from the bone microenvironment. For both MM and OS, the research assesses strengths, limitations, and data needs of current models, outlining the strategic objectives for next-generation multiscale, AI-enabled models providing a roadmap for tissue engineers, oncology scientists, and translational researchers to design clinically relevant preclinical tests and accelerate safer, more effective strategies for tumor-affected bones. The differences between MM and OS impose distinct biological constraints, so their comparisons are rare. Combining all these features with artificial intelligence capabilities will underpin a promising transition in the development of in silico adaptive and learning models.

## 1. Introduction

The spatial disposition and dynamic arrangement of cells govern their response to various factors and are fundamental to eliciting their functions [1]. For instance, bones are responsible for mechanical support, and also act as the cellular repository, being the source of both hematopoietic and osteogenic cells [2]. Bone marrow hosts hematopoietic stem cells that can develop into blood lineage cells and mesenchymal stem cells that generate osteogenic, chondrogenic, and adipogenic cell lineages while also supporting the marrow microenvironment [3]. Additionally, bones elicit some of the most fundamental immunological mechanisms that modulate homeostasis and engage in the pathophysiology of specific disorders. Stemming from their high plasticity and fast adaptive response, bones usually fulfill biological requirements, except under disease-related stimuli [4]. Still, regardless of their regenerative capability, bones remain a frequent site for cancerous conversion, demonstrating a delicate balance of their architectural, renewal, and immunological activities and their susceptibility to malignancy [5].

Considering the latest tactics of disease detection and therapy, bone cancers are still a continuing challenge for orthopedics and hematology–oncology [6]. Their tropism for either matrix or marrow is due to the abundance of cellular and molecular resources that sustain their proliferation and growth. Malignant development alters the homeostasis between bone resorption and regeneration, resulting in multifaceted neoplastic foci, notably lytic, blastic, or concurrent variants [7].

In oncology, in silico strategies are progressively establishing themselves as a cohesive connection among biological data, mechanistic understanding, and clinical decisional making, facilitating multiscale modeling, virtual trials, and precision therapeutics that underpin complex computational frameworks discussed in this research [8].

Multiple myeloma (MM) and osteosarcoma (OS) were selected among bone malignancies due to their significant clinical burden and persistent unmet need in medical practice. MM is the second most prevalent hematological cancer [9], with a rising global incidence and an incurable nature, as most patients experience relapses or develop resistance to the treatment [10]. More than 80% of patients suffer from osteolytic lesions, and approximately 50% get fractures, leading to a high mortality rate (between 20 and 24% of the cases) [11]. Although OS is less common than MM, it is the most frequent primary bone tumor and is associated with a 5-year overall survival rate that can be as low as 30% in patients with metastatic diseases, despite substantial therapeutic advances over recent decades [12,13]. Notwithstanding their distinct cellular origins, MM and OS demonstrate interconnected aspects of bone cancer, both characterized by bone tropism and osseous destruction, which highlight their molecular complementarity and malignant bone–microenvironment crosstalk [14,15]. By examining both malignancies concurrently, this review aims to identify computational opportunities by elucidating the overlapping mechanisms that drive tumor–bone interactions, thereby facilitating the discovery of emerging preclinical strategies.

Although other clinically significant bone malignancies (e.g., Ewing sarcoma [16], and chondrosarcoma [17]) along with hematological cancers (acute myeloid leukemia [18], chronic lymphocytic leukemia [19], and non-Hodgkin lymphoma [20]), are included in the large oncological landscape, our current focus is on MM and OS.

Thus, the aim of this research is to present a combined, computational perspective on multiple myeloma (MM) and osteosarcoma (OS), two biologically related but unevenly explored bone malignancies, by (i) comparing their molecular and microenvironmental characteristics, including signaling pathways and immune crosstalk; (ii) methodically assessing mechanistic, data-driven/machine learning (ML), hybrid, digital-twin/virtual cohort, and MIDD/PBPK frameworks developed for each cancer; and (iii) using cross-cancer comparison to recognize the shared and distinct biological and in silico axes. By structuring current models for MM and OS, bone-specific affinity, and common pathways, this research seeks to clarify the areas where the present strategies already reinforce oncology and where more comprehensive approaches are required. The present work constitutes a structured narrative comparative review.

## 2. Pathophysiology of MM and OS Relevant to In Silico Modeling

Bone cancers, namely, multiple myeloma (MM) and osteosarcoma (OS), exhibit dynamic mechanisms, shaping their behaviors and clinical outcomes. These two malignancies represent a molecularly heterogenous group of cancers unified by their mutual bone involvement and their dependence on the osseous microenvironment, yet they are markedly distinct in their cellular origin and clinical behavior [20]. In computational modeling, this variability presents both challenges and opportunities. The complexity of tumor biology requires multiscale in silico methods, while common pathological pathways across different diseases underpin the feasibility of generalizable modeling approaches [21]. Aberrant bone reorganization through central signaling pathways, dysregulated intracellular signaling cascades, cell survival and differentiation, the microenvironment dynamic crosstalk of bone marrow, and genomic instability are among the key pathophysiological features relevant to computational modeling [22,23]. In this context, MM and OS appear as two complementary entities with a largely distinct yet partially overlapping molecular landscape: MM as a plasma cell-derived hematological cancer and OS as a mesenchymal-origin solid tumor. This strongly associated dependence on the bone marrow niche and aggressive bone destruction makes them suitable models for comparative computational frameworks [24,25].

### 2.1. Multiple Myeloma

While comprising nearly 1% of all malignancies [26], MM accounts for approximately 10% of hematologic tumors [27], exposing its overrepresented impact and clinical relevance. Worldwide, MM impacts men at a higher frequency than women (55–57% of instances) and predominantly emerges in adults. The central age tendency is around 65–70-year-olds, followed closely by those aged 75 years, with a notable growth after 40 years [9]. The common sites of MM are the vertebrae and the pelvis area [10].

Multiple myeloma is characterized as a genetically heterogeneous neoplasm triggered in B cells [28]. The deviant clonal cellular expansion of plasma cells [29] resident in the bone marrow causes an overproduction of monoclonal immunoglobulin [30]. This phenomenon leads to organ malfunction along with bone lesions and additional anemia [31]. Atypical genomic rearrangements and hypermutations are driven by activation-induced cytidine deaminase (AICDA) [32]; this elicits DNA fragmentation that leads to instability and malignant transformation of B cells [10]. A substantial proportion of all MM cases evolve from an asymptomatic pre-malignant phase named monoclonal gammopathy of undetermined significance (MGUS) [33] or smoldering multiple myeloma (SMM) [34]. Some of the secondary injuries are related to chromosome alteration, notably deletion of 17p [del(17p)] or addition of 1q [gain(91q)] [35]. The sum of these abnormalities defines the therapeutic response and prognosis [9]. MM is influenced by both recurring activation of key intracellular pathways and cytokine signaling. Wingless/integrated (Wnt) signaling [36] and the phosphoinositide 3-kinase (PI3K) [37], protein kinase B (AKT) [38], and mechanistic target of rapamycin (mTOR) signaling axis pathways [39] are used to maintain survival, growth, and treatment tolerance [40].

The pathophysiology of MM is characterized by abnormal osteoclast activity [41]. Myeloma cells change the microenvironment, enhancing bone resorption while suppressing the osteoblast function. The receptor activator of nuclear factor *κ*b (RANK)/receptor activator of nuclear factor *κ*b ligand (RANKL)/osteoprotegerin (OPG) (RANK-RANKL-OPG) axis [23], the central regulatory axis of skeletal rehabilitation, drives osteoclast activation while osteoblast differentiation is impeded due to sclerostin, activin, and wnt-β-catenin pathway inhibition [42].

Pro-angiogenic growth factors (PAGFs) [43], such as vascular endothelial growth factors (VEGFs), fibroblastic GF (FGFs), and hepatocyte growth factors (HGFs), are synthesized and released in MM, supporting clonal expansion and therapy tolerance [44]. Finally, proinflammatory interleukins (ILs) [45] and transforming growth factors (TGF-ß) [46] amplify the imbalance between bone formation and bone resorption, leading to the genetic reprogramming of osteogenic mesenchymal progenitors [10]. In MM, cancerous plasma cells reset the marrow niche [39]. This pattern is characterized by aberrant natural killer (NK) cells, regulatory T-cell proliferation, T-cell fatigue, an increase in myeloid-derived suppressor cells, and macrophage polarization into malignant phenotypes [47].

### 2.2. Osteosarcoma

Osteosarcoma (OS) is a bone malignancy characterized by mesenchymal lineage conversion, in which abnormal osteogenic differentiation initiates aberrant tumorigenic osteoid production [48]. OS is the most prevalent primary osseous neoplasm [1]. With a 35% incidence of all malignant skeletal tumors, it displays two peaks in the age distributions. One peak coincides with fast skeletal development and pubertal hormonal fluctuations in adolescence (10–30 years old). The following peak is in late adulthood (60–65 years old) [49]. As in MM, this tumor has a male predominance [6]. Almost 75% of the affected individuals are under 25 years old. The most common sites of tumors are the metaphysis of long bones, especially around the knee and proximal humerus [50].

**Figure 1:**
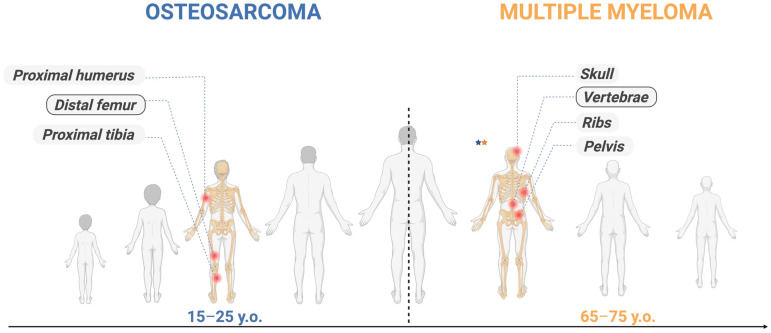
The evolutionary trajectory of an individual across the lifespan. Each malignancy has a color designation, with OS depicted in blue and MM in orange. The two neoplasms are situated on a timeline according to the age at which they most frequently arise throughout an individual’s existence. The red circle marks indicate the most common sites of each disease. The two asterisks (blue and orange) indicate that, to date, only three cases with the simultaneous emergence of MM and OS have been reported. Image created in BioRender. Ghita, A. (2026) https://BioRender.com/nqf64i2.

An illustration of the temporal mapping of selected neoplasms across the human ontogeny is depicted in Figure 1.

Intrinsic genetic shifts and extrinsic microenvironmental signals activate tumorigenesis through osteoblast proliferation [51]. While the steps of mesenchymal differentiation remain less clearly defined than those in the hematopoietic system, several transcription factors are now recognized as key regulators of mesenchymal cell fate. A SRY-box transcription factor 9 (SOX9) [52], osterix, and a runt-related transcription factor 2 (RUNX2) [53] are fundamental factors that indicate the osteogenic cellular profile. Their presence and dynamics are directly governed by tumor suppressors and oncogenes, such as tumor protein 53 (TP53) [54], MYC proto-oncogene [55], and bHLH transcription factor [56]. Additionally, major neoplastic pathways such as PI3K-AKT-mTOR [57], Hedgehog [58], wnt/β-catenin [59], Janus kinase-signal transducer and activator of transcription (JAK-STAT) [60], and NOTCH [61] are heterogeneously activated in OS. They vary greatly among cancers and constitute an integrated system that controls differentiation, stress adaptation, and growth [62]. A specific category of OS stem-like cells supported by NOTCH, Hedgehog, and wnt/β-catenin signaling are identified by the presence of SOX2, octamer-binding transcription factor 4 (OCT4) [58], nanog homeobox transcription factor (NANOG) [63], and Kruppel-like factor 4 (KLF4) [64], involved in therapeutic resistance and tumor relapse [65].

The tumor evolves from a multipotent mesenchymal stem cell and is characterized by malignant cells that generate an unmineralized bone matrix, with a vast population displaying adipogenic, fibroblastic, and chondroblastic phenotypes [48]. The interplay between cells and their milieu is crucial for neoplasm development and involves direct contact between vascular, mesenchymal, and immune cells. In the early stages of the disease, the tumor growth impairs the osteoblast–osteoclast balance, mobilizing pro-tumorigenic factors [66]. OS cells with coexisting marrow populations reshape the architecture of the entire bone, producing the carcinogenic osteoid. The altered osteoid extracellular matrix stimulates malignant growth, invasion, and drug tolerance, promoting metastatic spread [67]. In this area, regardless of progress in therapy, outcomes stagnated. Yet, new approaches to tumor biology yield some promising results through molecular pathways and antigens [68]. OS has a substantial predilection for pulmonary metastasis. Its microenvironment is abundant in M2-like tumor-associated macrophages (TAM2) [69], coupled with C-C motif chemokine ligand 18 (CCL18) [70] and cyclooxygenase-2 phosphorylated signaling (COX2/p-STAT3) [71], correlated with cancer dissemination and a guarded prognosis [72]. This is an essential determinant of patient survival and remains a clinical challenge. Malignant cells guide mesenchymal stem cells (MSCs) via extracellular vesicles with tumor growth factor β (TGF-β), triggering Interleukin-6 (IL-6) production and activating a cascade that initiates metastatic lesions in the lung [73]. Extracellular vesicles from OS cells reset myofibroblasts and stem cells to metastatic seeding [74].

### 2.3. Shared and Distinct Biological Axes

Although MM and OS are distinct, they share some common features, including RANK/RANKL/OPG activity, the PI3K-AKT-mTOR pathway, Wnt signaling, TGF-β tone, and IL-6/STAT3. MM is additionally marked by del(17p) and RUNX2 imbalance, while OS reveals TP53 loss, SOX9, JAK-STAT, NOTCH, and Hedgehog aberrant activation [75,76].

MM is characterized by a gradual genomic heterogeneity, with successive AID mutations, chymotrypsin rearrangement, and MYC activation [77]. OS features chromosomal instability, with TP53 and RB1 variations and chymotrypsin rearrangement [76]. In terms of genomic instability, these two malignancies share genomic instability via distinct patterns.

From an evolutionary point, MM commonly arises from MGUS/SMM. This early transcriptional alteration enables ribonucleic acid sequencing (RNA-seq), indicating clonal expansion [77]. In OS, the RNA-seq quantifies gene expression and can be used to infer cancerous stem cell-like (CSC) states through stemness-associated transcriptional signatures, in a temporal pattern [78]. Therefore, both cancers share evolutionary variation with stem-cell-like properties originating from mesenchymal or plasma-cell populations.

In the tumor microenvironment (TME) and immune reprogramming, MM actively reorganizes the bone marrow (BM) through CD8 cytotoxic T lymphocytes (CD8 T-cells), macrophages, and ligand–receptor pathways, such as HGF, macrophage migration inhibitory factor (MIF), IL-15, and B-cell activating factor (BAFF), from MGUS to MM [79]. OS development is induced by M2 TAMs triggered by hypoxia, STAT3/NF-kB/ERK signaling, and VEGF/FGF2-AKT angiogenesis. Single-cell transcriptomics indicates neoplastic clusters that release Wnt, TGF-β, and bone morphogenetic protein (BMP) ligands [80]. Across MM and OS, neoplastic dynamics are mediated by TME. Vascular pathways and stress stimuli govern the conditions, although they arise in different bone niches.

**Figure 2:**
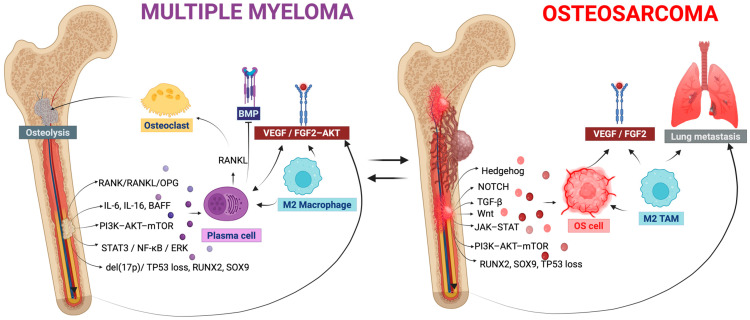
Schematic illustration of bone-centered disease dynamics for MM and OS with common and specific features. MM develops from malignant plasma cells within the hematological niche and is driven by tumor–bone–immune interaction, such as RANK/RANKL-mediated osteolysis, cytokine signaling, and NF-kB pathways. OS originates from osteoblastic cells in the bone matrix and is controlled by oncogenic pathways such as NOTCH, Hedgehog, or JAK–STAT. Both diseases share PI3K-AKT-mTOR and pro-angiogenic factors such as VEGF/FHF2 that sustain the tumor growth and bone remodeling. TP53, RUNX2, and SOX9 are present in both malignancies, with disease-specific roles. M2 tumor-associated macrophages occur in MM and OS, creating a pro-tumoral microenvironment. In MM they support plasma-cell survival with altered bone morphogenetic protein signaling (BMP), tumorigenesis, and osteolysis, while in OS they enhance angiogenesis and metastatic dissemination. This figure provides key features that might motivate common computational and AI modeling strategies. Image created in BioRender. Ghita, A. (2026) https://BioRender.com/4qfrcqs.

Figure 2 depicts the MM and OS biological networks and data context.

## 3. In Silico Modeling Frameworks for MM and OS

Considering the interconnected heterogeneity of MM and OS, namely, clonal dynamics, altered bone reorganization, and interconnected tumor–bone–immune niches, computational frameworks provide a safe strategy to assess challenging hypotheses for both preclinical (in silico, in vitro, ex vivo, and in vivo) and clinical scenarios [81,82]. The following section addresses five computational paradigms applied to MM and OS within the cross-disease framework developed in this review.

### 3.1. Mechanistic Models

Mechanistic models emphasize present biological, chemical, or physical processes, e.g., tumor growth dynamics or pharmacological modulation, as equations, enabling a personalized, patient-specific approach [83]. These frameworks combine deterministic or stochastic representations rooted in biological architecture, such as quantitative systems pharmacology (QSP), and models employing ordinary differential equations (ODEs) and partial differential equations (PDEs) for neoplastic expansion, osseous reorganization, or immune-mediated cancer interactions. Mechanistic frameworks are implemented for causal explanation or hypothesis synthesis. In MM and OS, such models were employed to address bone–tumor crosstalk, such as RANK-RANKL-OPG signaling, clonal proliferation, therapeutic outcome, or growth factors in the bone marrow milieu [82]. The models developed for MM address marrow-level kinetics and cytokine feedback, along with the progression from precursor stages to active MM, justifying their inclusion in this classification [84,85,86]. Alternatively, mechanistic hypotheses of osteoblast–osteoclast dynamics, osteoid spatial proliferation, and metastatic spread are extensively validated for OS [87,88].

### 3.2. Data-Driven/Machine-Learning (ML) Models

In these models, extensive scientific data resources and predictive algorithms are implemented to identify patterns and generate predictions with context-dependent mechanical assumptions [89]. Data-driven frameworks, e.g., classical ML and deep learning, are implemented to facilitate quantitative prediction but lack causal interpretability. Machine and statistical learning, pharmacokinetics (PK), and response-predictive designs are compatible with this modeling type. Lacking a clear mechanistic design, these schemes instantly recognize patterns in clinical, imaging, molecular, or histopathological data. Each of the two considered malignancies provides representative examples in therapeutic responses, outcome forecasting, and multi-omics subgrouping [90]. For MM, this approach can predict the overall survival time or therapeutic outcome from the real-world data. For OS, besides forecasting, ML frameworks support radiomics-based diagnosis, staging, and metastasis occurrence [91].

### 3.3. Hybrid Mechanistic-Learning Models

This approach employs a hybrid or mechanistic-learning framework that combines the strengths of a mechanistic design with elements of data-driven schemes to optimize forecast efficiency, for example, for bone remodeling and tumor growth [92]. The core mechanistic components are expressly defined, quantitatively specified and data-constrained, or expanded by learning-based models; insufficient data availability can affect their predictive capability. The hybrid design results in a distinct modeling paradigm. Both shallow and deep integration can be used as modeling approaches. In the former framework, ML and mechanistic designs are merged gradually, while in the latter, the computational data are integrated directly or collaboratively refined [93]. Hybrid MM models commonly integrate mechanistic features of osseous-tumor dynamics with statistical data obtained from patients.

Such frameworks are implemented for causal explanation or hypothesis synthesis. For instance, the mathematical ODE framework (a computational model that describes the capability of a system to change over time based on tracking the state variables) characterizes how malignant cells and osteoclast crosstalk are calibrated to individual biomarker specificity [94]. An OS framework comprises osseous remodeling equations enriched with data-driven features to assess signaling pathways [95].

### 3.4. Digital-Twin/Virtual Cohort/“In Silico Clinical Trial” Models

This type of models builds by formulating a virtual patient or cohort trial to simulate a clinical trial result, thereby reducing reliance on experimental studies [96]. This approach is not based on a model but on a dynamically recalibrated, patient-tailored parameterization method enhanced with longitudinal data inputs [97]. To the best of our knowledge, such frameworks are currently relatively uncommon and conceptually different from static models. For MM, such models were implemented to a greater extent and combine longitudinal measurements of M-protein levels, therapy graphics, or skeletal markers to assess patterns of disease progression and relapse. For OS, this approach is mostly conceptual, using limited pilot studies. Some designs employ patient-tailored neoplastic growth models parametrized to imaging data with the aim to predict chemotherapeutic responses [98]. A virtual comparison between MM and OS on overlapping pathways, including RANK-RANKL-OPG signaling and PI3K/AKT/mTOR activation, can demonstrate how bone–tumor networks react to equivalent therapies [99,100].

### 3.5. Model-Informed Drug Development (MIDD)/Physiologically Based Pharmacokinetic-Type (PBPK) Models

Models of this type rely on computational frameworks (commonly pharmacokinetics/pharmacodynamics or patient-oriented) to establish doses, risk avoidance, and efficient early developmental decisions [101]. MIDD designs are defined by collective application context, namely, regulatory applicability, decision assistance, or translational relevance, rather than mathematical structure. In modern models, QST (quantitative systems toxicology), QSP, and ODE systems, pharmacokinetic/pharmacodynamic (PK/PD) designs, Bayesian frameworks (computational schemes that rely on applying probability to describe uncertainty while refining their data whenever novel knowledge arises), and ML methods can be used simultaneously [102]. PBPK designs define therapeutic absorption, drug metabolism, and clearance employing bone-tailored, anatomically accurate sites [103]. Together, they can calibrate the drug dosage, drug–drug interactions, assess risk–benefit outcomes, or design robust clinical trials [104,105,106].

**Figure 3:**
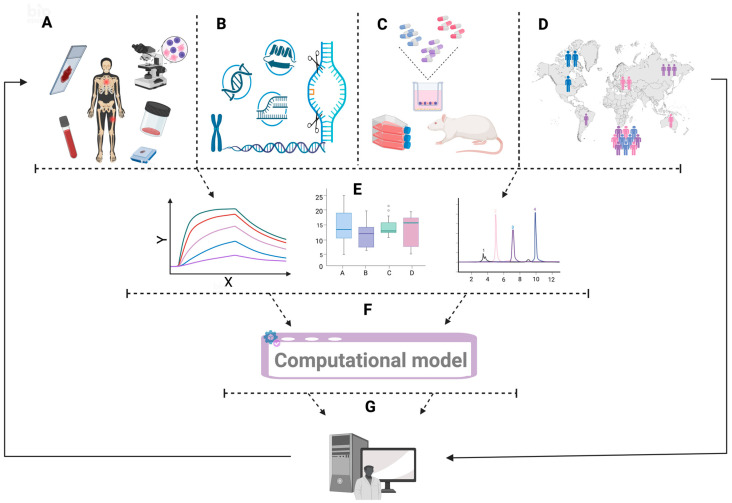
Integrated preclinical and clinical dataset for generation of multidimensional in silico records for OS and MM. It illustrates the data integration into the construction of computational models for MM and OS. Such research originates from the comprehensive collection of tumor-specific information, including localization, clinical evolution, imaging results, and histopathological characteristics (**A**). These data are merged with higher-resolution molecular profiles generated with genomics, transcriptomics, proteomics, and related platforms (**B**). Once integrated, they guide preclinical experimentation in representative in vitro systems and in vivo models to validate the biological relevance of the initial observations (**C**). The final step involves global consolidation of these datasets and their translation into in silico frameworks, allowing quantitative reconstruction of tumor dynamics and prediction of therapeutic responses (**D**). The process remains iterative: each dataset reshapes a new model (**E**), which, in turn, guides further data collection (**F**), creating a cycle of refinement (**G**) rather than a finite endpoint. Image created in BioRender. Ghita, A. (2026) https://BioRender.com/avsf1on.

A conceptual framework that clarifies the integration and propagation of knowledge across the scales is necessary to justify the input of multicomponent data into computational disease modeling. Figure 3 presents such a system for in silico models.

## 4. Perspective of Cross-Cancer Comparison: MM Versus OS

Cross-cancer comparison augments the comprehension of the diseases’ patterns and therapeutic approaches. It should be stated here that the computational paradigms addressed above are disease-agnostic and analyze two physiologically different osseous neoplasms. MM and OS are typically approached, parameterized, and treated as independent entities. They vary in cell origin, clinical pathway, and microenvironmental interplay. In silico models addressed are, therefore, an opportunity to recognize the features that might be illustrated for these tumors without assuming a singular disease narrative.

After examining each modeling paradigm separately in Section 3, this part summarizes the cross-disease computational insights that arise through comparative application of the unified framework across MM and OS.

### Computational Translations and Cross-Disease Insights

Mechanistic models of cancers add value through their causal structure encoding neoplastic and milieu frameworks that are optimal for RANKL-pathway osteolysis characterization, angiogenesis, or PI3K-AKT-mTOR signaling [107]. MM benefits from robust data on the bone marrow niche, enabling detailed multiscale tumor–bone–immune designs. This serves as the foundation for digital-twin models of MM [100]. In OS, the strength of such models stems from the well-defined pathways of knowledge, allowing analogous multiscale frameworks. One constraint of this scheme for OS is that the bone marrow compartment and immune modulators are poorly developed [108].

Data-driven and ML designs provide pathway patterns from clinical, omics (genomics, transcriptomics, proteomics, and epigenomics), and imaging knowledge without detailing the underlying software implementation [67,109]. For example, in MM, gene-expression-framed ML metrics categorize the subjects and predict the outcomes based on del(17p) and NK-kB biology. For OS, the strengths of this approach lie in radiomics based on computed tomography (CT), magnetic resonance imaging (MRI), positron emission tomography (PET), and deep learning. It forecasts therapeutic outcomes, diagnoses, or metastasis by merging VEGF/FGF2-AKT, BMP, and TME reorganization detected in imaging [110]. The limitation of this model are reduced cohorts, especially for OS, diminished interpretability, and low connection to explicit pathways.

Hybrid mechanistic-learning models provide a feasible strategy for ML to optimize bone-niche QSP frameworks, utilizing biological models, such as M2 TAMS or ILs, to predict multi-omics and therapeutic outcomes for patients [107]. For OS, the potential emerges from the reutilization of MM modules while allowing ML optimization of OS-specific patterns [111].

Digital twins and virtual cohorts offer value by supporting cross-oncology evidence [110]. In terms of MM, the strength is thanks to robust longitudinal data and validated QSP designs [96]. For OS, the rarity and heterogeneity of the disease provide an amenable background for digital-twin frameworks. One specific example may be the combination of this design and pan-cancer histopathology foundation approaches, such as Virchow’s. The Virchow model is an AI tool that explores limited data availability in rare cancers [112].

MIDD/PBPK models, employing AI-enhanced pharmacometric designs, enrich the computational approaches by translating data from niche biology and pathways to therapeutic outcomes, dose parameterization, or exposure responses. The value of this approach is its demonstration that ML models can support derivation of the PK/PD variables and ability to reinforce digital-twin models in terms of treatment-related toxicity and clinical efficacy across oncologic diseases [103].

The recent data [113] indicate that MM has an extended single-cell immunological database and longitudinal multi-omics, incorporating also large cohorts. Multi-omics and single-cell integrative research have also become more prevalent for OS, including integrative modeling that combines omics elements. Modern computational schemes supported by AI, such as large language models (LLMs) (data-driven neural frameworks used in probabilistic language formulation), can leverage these similarities to promote cross-disease optimization [107,114].

**Figure 4:**
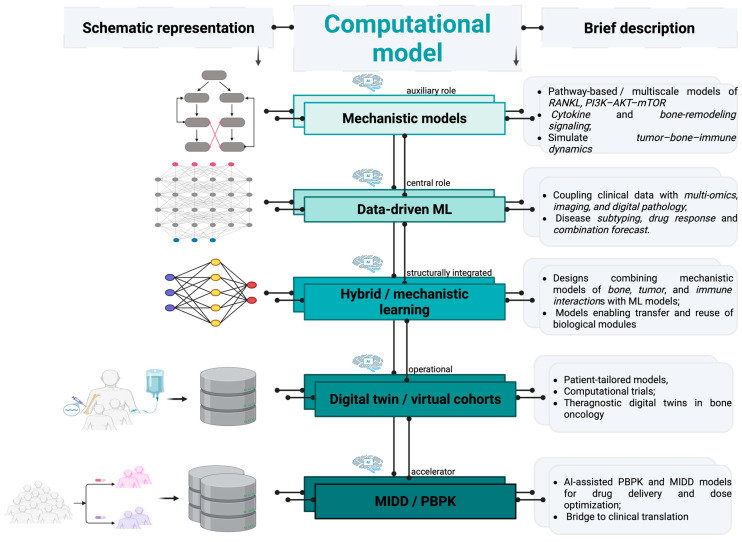
Computational frameworks for MM and OS with shared features. Mechanistic models address pathway-based and multiscale tumor–bone–immunity interplay, with AI serving an auxiliary role. Data-driven machine-learning frameworks incorporate multi-omics data with imaging and digital pathology while also simulating drug outcomes. Hybrid mechanistic–ML designs couple mechanistic features with machine learning datasets in terms of tumor dynamics, bone interplay, and immune interactions. AI is structurally integrated in these models and can transfer and reuse biological modules between MM and OS. Digital-twin and virtual cohorts implement patient-tailored models for trials and theragnostic applications, with AI having an operational role. PKPB and MIDD approaches collect data from patients, enhance the pharmacokinetics, optimize dosage, and create a link between preclinical and clinical research. Image created in BioRender. Ghita, A. (2026) https://BioRender.com/n96g9td.

Figure 4 illustrates computational models integrating inputs from multiple myeloma and osteosarcoma.

Table 1 summarizes the common and distinct features relevant to computational modeling of MM and OS.

**Figure 5:**
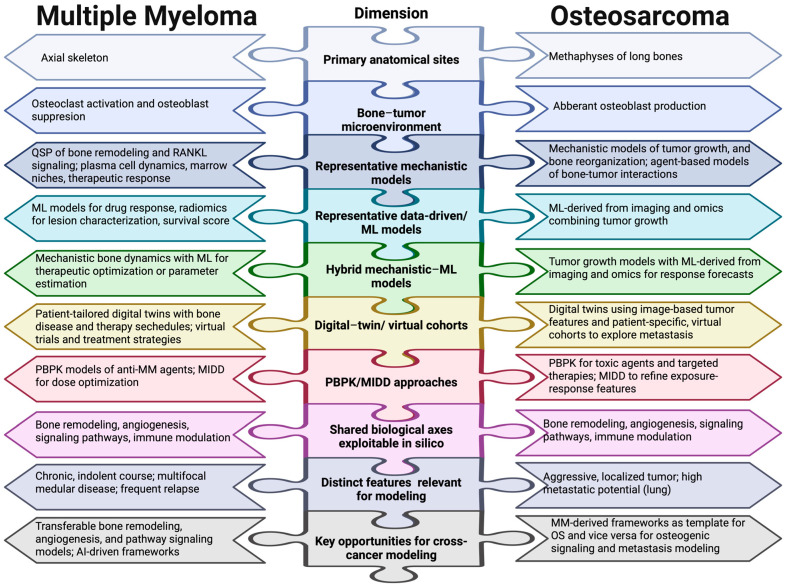
Comparative summary of MM and OS and their computational frameworks. The overview outlines biological features as well as in silico commonalities and distinctions between the two malignancies, highlighting their applicability for computational modeling. The tumors differ significantly in the age of onset, skeletal distribution, and disease dynamics. Nevertheless, a few common features are identified, namely, angiogenesis, pathway signaling, and bone remodeling. Shared dependency provides a common biological feature that may be also exploited. Image created in BioRender. Ghita, A. (2026) https://BioRender.com/ju9ddcm.

Table 1 provides a summary of features and approaches for MM aligned with OS among essential biological and computational dimensions. It correlates oncogenic networks, bone niches, immune and cytokine microenvironments, and datasets with their specific characteristics and current limitations. Figure 5 offers an illustration of the same dimensions for two distinct malignancies.

This schematic representation offers a side-by-side overview of MM and OS across ten key areas with high relevance for computational modeling. Pathophysiologically, osteoclast activation and osteoblast inhibition are the main pathways in which MM impacts the bone, affecting the axial skeleton [23]. Conversely, abnormal osteoblast synthesis triggers OS to arise in the metaphysis of long bones [22]. These malignancies represent two types of osseous dysregulation that are mechanistically different yet computationally convergent. From a modeling perspective, illustrative mechanistic frameworks for MM consist of Quantitative Systems Pharmacology models of bone reorganization and RANKL signaling [86]. Agent-based approaches of bone–tumor dynamics and tumor progression were used to address OS [88]. Therapeutic response prediction and radiomics-based lesion characterization in MM both benefited from the application of data-driven and ML models [90], as did imaging and omics integration for the modeling of OS [89]. Hybrid mechanistic–ML models, digital-twin approaches, and PBPK/MIDD strategies were established independently for each malignancy [96,136]. Most significantly, bone remodeling, angiogenesis, immunological regulation, and signaling pathways are exploitable axes shared by both illnesses, serving as the common computational ground for cross-disease modeling [21]. As previously mentioned, their unique features—the aggressive, localized, highly metastatic behavior of OS against the chronic, multifocal, relapsing course of MM—define the parameters of this comparison and influence the transferability of modeling frameworks between the two illnesses.

## 5. Discussion

In silico models represent a novel, methodological advance approach for simulation and assessment of tumor–bone interplay, prediction of therapeutic outcomes, and optimization of preclinical framework design. Such computational studies can deliver quantitative insights into illness progression, microenvironment dynamics, and therapeutic efficacy while reducing reliance on conventional in vitro and in vivo experimental models [137]. These designs offer a safe, cost-effective, and time-efficient alternative that minimizes the need for animal or human testing.

In the current research landscape, artificial intelligence (AI) has evolved as a cognitive platform, redefining how in silico models are developed. Still, AI does not establish new models; its impact is on the mechanistic function of the current frameworks by redefining interconnections between the model’s design, behavior, and reliability [98,138,139].

### 5.1. Critical Appraisal of Current Computational Approaches for MM and OS

Mechanistic, data-driven, and hybrid approaches have evolved significantly in oncologic research. Still, MM and OS continue to expose critical knowledge gaps. Mechanistic ODE/QSP/PKPD models include the established signaling cascades and drug–tumor interplay. For instance, QSP and PBPK designs regularly encode PI3K-AKT-mTOR and associated tumorigenic networks and are substantially used in MIDD. They can forecast efficacy and dose parameterization as well as simulate digital trials in bone malignancies [40,140]. For MM, computational and 3D models of the bone marrow niche simulate stromal adhesion, hypoxia, cytokine dynamics, and osteoblast–osteoclast interplay, incorporating the role of the PI3K inhibitory effect on bone resorption and remodeling [141]. Mechanistic immuno-oncology QSP frameworks consider elements of the tumor immunity cycle and are employed as a key checkpoint regulating T-cell activity in programmed death-1 (PD-(L)1) combinations among numerous cancers [100]. In 2023, Urdeitx et al. [142] described an agent-based/finite-element approach that integrated cell motility, proliferation, apoptosis, and signaling interactions of original bone tissue and stromal cells but in the MM-like context. Existing Wnt-mediated stromal cell signaling, and a detailed mechanistic description of the skeletal microenvironment hypothesized by Belik et al. [143] in the MM context are increasingly integrated into bone cancer models, namely, for OS. These frameworks outperform pathway-based mechanistic reasoning and experimental tests exploring inhibition strategies or niche-targeted regimens before the clinical assessment [144,145].

Despite their advanced complexity, important limitations persist in the use of mechanistic models. Immune-oncologic QSP frameworks face constraints with intra-tumoral heterogeneity of T-cell density and cytokines [146]. Comparative research demonstrates that ODE-based models can recreate dynamics yet fail to address spatial organization. The lack of these features is critical in immune checkpoint inhibitors that agent-based models may consider [147]. Clonal evolution and therapy tolerance reveal heterogeneous trajectories in MM with dysregulated metabolism and immune evasion post-treatment [148]. Furthermore, 3D myeloma frameworks identify distinct microenvironmental sensitivities in terms of extramedullary malignancy versus bone-marrow-restricted clones. Most models use simplistic interactions and cannot quantify robustly the involved mechanisms [149]. Regardless of their biological interpretability and capability to formulate testable hypotheses, numerous models were validated computationally, with limited independent experimental or clinical validation, which reduces confidence in their predictive accuracy [85,87], raising questions regarding repeatability and generalizability [107].

In contrast, data-driven ML and AI approaches have a significant value in analysis of skeletal cancers. ML designs, calibrated on radiomics, histopathology, or multi-omics, provide important data on biomarker discovery or immune milieu characteristics from digital slides. However, they rely strongly on very large, well-annotated datasets while being affected by limited interpretability [140]. These models have difficulty representing the pathway structures, such as RANK-RANKL-OPG, Wnt, or NOTCH, and extrapolating beyond the observed therapeutic regimens [150]. Liquid-biopsy-driven ML approaches describe circulating cell-free tumor DNA kinetics, yet they do not integrate the bone niche reorganization. These strategies are pan-cancer approaches applicable to solid tumors, such as OS [151]. Although ML frameworks for both malignancies showed promising discriminative outcomes, most of them were developed on small, single-institution cohorts. Without external validation and insufficient compliance with predetermined reporting guidelines, their reproducibility remains limited [152,153].

Analytical reviews reveal a small number of real hybrid models, where mechanistic frameworks and ML are highly interconnected [82], particularly those that combine MM and OS. ML enables estimation of, and introduction of constraints for, parameters with limited measurability, such as tumor immune infiltration or cell-state fractions from imaging data or single-cell knowledge. This information is further integrated into a mechanistic model, and the therapy simulation is initiated [154]. Mechanistic designs can also derive digital cohorts or virtual patients to amplify the limited clinical datasets for training of ML predictors [155]. Despite this, hybrid models continue to be technically demanding, constrained by data harmonization, with limited prospects of their validation [156]. The degree to which the mechanistic component restricts model behavior instead of serving as a regularization layer is rarely evaluated through ablation analysis. A standardized validation structure for this class of models in oncology has yet to be clearly defined [82,111].

Digital-twin and virtual-patient frameworks represent an evolving paradigm, remaining mostly experimental. In 2024, an evidence mapping review in oncology identified only 30 digital-twin studies [157]. Most of them utilized synthetic or low-sample datasets, with limited clinical validation. A real-time knowledge or ML-driven customization was scarcely observed [158]. Theoretical and regulatory systems highlight their anticipated value in terms of trial parameterization and support of patient decision-making. However, they have deficiencies in data integration and credibility assessment [159]. Although conceptually promising, numerous digital-twin models for MM addressed here remain at the proof-of-concept stage. They are validated retrospectively on small patient cohorts without prospective evaluation against real clinical results. For OS, a dedicated digital-twin framework remains underdeveloped, underscoring the gap between their computational readiness and clinical requirements [100,138].

MIDD, PBPK, and semi-mechanistic PK-PD models provide a foundation for dose selection and schedule optimization in oncology research. They characterize the organ-level drug distribution and time exposure [107,160]. These designs usually address the biology and development of tumors in a simple manner [161], being insufficiently focused on MM or OS niche dynamics or immune modulation. With limited effectiveness for long-term tolerance patterns, high-level system models are required [162]. PBPK and MIDD approaches remain some of the most direct clinical translations among the frameworks addressed in this research. Still, they also have some limitations: MM models rely on pharmacokinetic parameters derived from early-phase datasets and may not reflect all patients’ outcomes [163]. Meanwhile, in OS, methotrexate models were developed for pediatric cohorts with limited applicability to adult patients [107].

Considering that MM and OS display an osteoclast-mediated bone loss, mechanistic and quantitative frameworks based on the RANK/RANKL/OPG-axis established for MM offer some of the most readily transferable approaches from MM to OS [164]. Similarly, PBPK and MIDD approaches validated for MM anti-resorptive agents may be adapted to optimize dosing in OS, where such frameworks remain at an early stage of development [165], particularly in addressing the pharmacokinetic and pharmacodynamic challenges presented by pediatric patients.

Radiomics-based ML frameworks for response predictions can be transferred from OS to MM thanks to the similar nature of osteolytic lesions and the increasing use of imaging for disease monitoring. Regarding neoadjuvant chemotherapy responses in OS, these processes achieved good accuracy. As a result, they may be included in models for MM imaging responses [166,167]. Additionally, current studies are focused on the use of 3D, agent-based, or spatial models to replicate the OS tumor microenvironment architecture. The development of MM bone marrow niche frameworks can be influenced by these models. This can be especially important to illustrate the spatial dynamic among immune populations, stromal components, and plasma cells [146,168].

Overall, for MM and OS, current computational models robustly encode signaling pathways and the bone microenvironment. Still, they underrepresent complex immune cell dynamics, exemplified through Treg cells, myeloid populations, or CD8+ T cells [116,169], or therapy-driven trajectory and metastatic transitions [170,171,172].

### 5.2. Integrating AI and Multimodal Data Across MM and OS

Recent available data on multimodal deep learning demonstrate that MM and OS frameworks can shift from single-mechanism paradigms by merging omics, pathology, medical imaging, and clinical data into a unified patient representation [173]. Recent reviews of the use of artificial intelligence for biomarker identification and immune checkpoint inhibitors (ICIs) stress that single dataset types, namely, proteomics, genomics, and radiomics, partially represent tumor heterogeneity [174]. Recent models can support MM and OS approaches with rich multimodal AI layers [175].

Large-scale foundation frameworks, namely, Virchow [113] or PathOrchestra, indicate that self-supervised training of a substantial number of whole-slide images produces generalizable slide embeddings for pan-cancer or rare-cancer detection, biomarker forecasting, and reconstruction of gene expression profiles [113]. The transformer-based pathology Image and Text Alignment Network (TITAN) [176] expands this knowledge to the multimodal pathology–image–text alignment, facilitating identification of rare cancers, survival forecasts, and report generation with limited labeled data. Key features like these may be adopted in MM/OS digital twins as pathology encoders, modeling bone microenvironments and immune patterns that recent mechanistic models represent with limited fidelity.

Throughout oncology, ML systems are commonly used to guide prognostic and predictive biomarkers. Multi-level knowledge outperforms single omics but requires complex ML tools [177]. Frameworks such as ML-based consensus prognostic signature (MLPS) [178] or AI-driven multi-omics profiling [179] could identify niche-dependent mechanisms for common constraints. Flexynesis [180], a deep learning tool for precision oncology, lies at the foundation of advanced models. In MM, radiomics, AI, PET/CT and MRI previously linked imaging with bone tumor mechanisms to improve forecasts and therapeutic outcomes and to create powerful digital-twin models [181]. Radiomics and AI also supported the OS models to predict neoadjuvant chemotherapy and patient outcomes [166]. In OS, the Artificial Intelligence-Derived Prognostic Index (AIDPI) [182] provides an efficient example of AI integration that can identify abnormal metabolic and signaling pathways, namely PI3K-AKT-mTOR, supporting the generation of mechanism-informed therapeutic hypotheses.

### 5.3. Positioning Digital Twins and Virtual Cohorts for MM and OS

Population (pop) PK/PD and PBPK frameworks mostly quantify the absorption, distribution, metabolism, and excretion (ADME) processes [183] without considering specific individuals. Based on them, popPK/PD designs for MM [163] and OS [160] play an established role. According to this research, virtual cohorts/patients correspond to ensembles of mechanistically customized individuals. Their features can be depicted from clinical or omics datasets [184]. For individual patients, digital twins provide dynamic, continuous, and bidirectional connections with a physical person optimized with multimodal disease data, such as CERTAINTY [185], a pioneering virtual twin used for personalized cancer treatment in CAR-T-cell therapy for MM. Theragnostic digital twins (TDTs) [186] provide an example of bidirectional updating by integrating PBPK approaches with radiopharmaceutical features into a patient-tailored framework that allows optimization of agent selection. This type of refined concept can also be implemented in MM and OS modeling.

MM and OS are promising illnesses for evaluation of these methods thanks to their adequate anatomical characterization and medical imaging, allowing strong analysis at the population level. At the same time, MM is a heterogeneous disease, while OS is a rare malignancy, leading to their compatibility in digital-twin and virtual-cohort modeling.

### 5.4. Limitations of Current AI and Hybrid Models for MM and OS

Most AI cancer models are developed on limited, single-centered, retrospective cohorts, with limited population-based diversity, resulting in model overfitting and a lack of external validity [187]. For MM, radiomics and AI prognostic models were trained on less than 100 patients, so the outcomes were considered preliminary and not yet applicable in standard practice [188]. MRI-based 3D convolutional neural network (3D-CNN) risk frameworks are constrained to modest single-country datasets, highlighting concerns of unreliable calibration [189]. For OS, several radiomic ML models are developed on small cohorts, demanding multicenter validation prior to clinical implementation [182].

Deep learning frameworks for oncologic imaging and biomarker identification commonly lack transparency or biological plausibility. This methodological opacity raises concerns about safety, including unnecessary therapeutic augmentation for MM or high-toxicity protocols for OS [190,191].

The conceptual and methodological inability to accurately distinguish between prognostic and predictive signals is another issue that is addressed. Numerous studies explore therapeutic cohorts before employing trial-like methods, then considering any result-associated trait as “predictive”, even if it could just represent baseline risk [166,192].

As discussed, several established models exhibit methodological flaws, especially in validation and reporting, which might compromise their therapeutic usefulness, according to the available research [193,194]. Analysis of advantages and limitations of computational models is given in Table 2.

### 5.5. Toward Next-Generation MM and OS AI

Recent MM and OS approaches are moving toward AI-driven, multi-omics-integrated precision oncology, supporting more accurate prognosis and treatment selection [134,199,206].

Transparent Reporting of a Multivariable Prediction Model for Individual Prognosis or Diagnosis-AI (TRIPOD + AI) [153], Consolidated Standards of Reporting Trials-AI (CONSORT-AI) [154], and Standard Protocol Items: Recommendations for Interventional Trials-AI (SPIRIT-AI) [207] represent three international reporting standards integrating artificial intelligence in clinical research. Application of these reporting guidelines and validation criteria to MM and OS can be critical for bias mitigation and performance assurance, uncovering nonlinear configurations that remain invisible to conventional models.

COMpass [208], a geometric graph neural network (GGNN), provides another next-generation computational model utilized for MM that surpasses established methods. This approach adds value by integrating multi-omics data with pathway networks to predict survival.

Mistral-7B + retrieval-augmented generation (RAG) [209], a specific model built for MM, integrates RNA-seq, genetics, and clinical knowledge, refining patient-tailored therapeutic models.

In the area of OS, one specific AI-driven pathway-anchored model is the AIDPI framework, based on multi-algorithm ML, that was suggested to expose metabolic vulnerabilities and provide pathway-optimized therapies [182].

### 5.6. Limitations of This Review

To adequately contextualize the future perspectives and conclusions with the rapidly evolving research on bone cancers and computational oncology, it is necessary to indicate some of the limitations encountered in this review.

First, biological knowledge, molecular features, and pathophysiological frameworks relate specifically to MM and OS. Therefore, the generalizability of the findings to other bone malignancies cannot be assumed without disease-tailored validation.

Second, the computational approaches were reviewed in the context of MM and OS. This constraint reflects a deliberate attempt to maintain analytical coherence within a unified computational approach. As a result, other primary bone malignancies or hematological cancers may differ and require independent assessment before extrapolation.

A third limitation reflects the imbalanced availability and maturity of data resources. While MM is supported by high-dimensional omics data and harmonized clinical-genomic cohorts, OS datasets remain relatively small, heterogeneous, and less standardized. Consequently, certain aspects of the comparative approaches remain better suitable for MM than OS. This also represents the main justification for this cross-disease comparative study and a topic for additional research in addressing the computational and biological differences between MM and OS.

## 6. Future Perspectives

This rapidly developing field still faces considerable challenges that will define its future progress. In mechanistic modeling, there is still limited data availability, particularly for OS. Hybrid data fusion with ML and AI can optimize the performance of models in terms of parameter estimation, calibration, and predictive reliability [83].

With respect to the data-driven/ML models, multimodal deep learning combining omics with imaging and pathological features may be a novel approach [109].

Digital twins and virtual cohorts face a limitation in terms of standardized multi-omics, imaging, or epidemiologic datasets. Ethical and privacy concerns should be also addressed, along with validation constraints. Future developments may take into consideration a dynamic framework with data fusion that provides a recurrent parameter updating [210].

For MIDD and PBPK models with AI capabilities, LLMs may offer an assistive approach throughout pharmacometrics modeling designs, consolidating a hybrid mechanistic–human–AI direction [211,212].

From another perspective, MM and OS models can be re-architected from distinct pathologies. One example may be bladder cancer models [213], by creating an analogous application of a graph-based multimodal framework integrating a hematoxylin and eosin (H&E) histopathological stain (for cellular and tumor morphology) and RNA sequencing (to quantify gene expression). Another example may be from HPV-positive oropharyngeal cancer [214]. Despite being validated for another tumor, it may permit the transfer of disease-tailored features from CT/MRI radiomics and histopathological examination of the tumor while retaining cross-modal features.

A highly promising area of inquiry for MM and OS is the integration of spatial transcriptomics (ST) data into in silico modeling. This ST approach maintains tissue architecture and cell–cell dynamics, the key features of tumorigenesis and progression [215,216]. By incorporating such spatially resolved multi-omics, a feasible approach may clearly encode tumor–microenvironment heterogeneity or metastasis interconnections in MM and OS.

Given the fact that MM and OS have smaller cohort sizes than typical epithelial malignancies, the development of foundation models pre-trained on extensive multi-omics and imaging data may be pertinent. Through transfer learning, these self-supervised models built on large data may be tailored to illnesses, allowing for reliable predictions and biological understanding even in malignancies with limited datasets [217,218,219].

A relatively understudied yet feasible direction for OS is cross-species computational approaches that couple human, canine, and murine transcriptomic datasets. A rationale for adopting such models is the similarity of genetics, histology, and metastatic patterns in both species while canine OS is more common than human OS [220,221]. Embedding these cross-species cellular and microenvironmental datasets into computational approaches may provide a key method to validate spontaneous OS from dog models and genetically engineered murine OS with human models [222].

A potential yet understudied strategy for MM and OS may be the generation of a multiscale digital-twin model that integrates patient-tailored, tissue-level bone biomechanics with molecular and cellular disease models. Both malignancies are associated with pathological fractures resulting from the changing mechanical competency of osseous tissues driven by bone remodeling [223,224]. Integrating such approaches in schemes for MM and OS by combining mechanobiological models with oncologic digital-twin designs may enable clinical predictions, such as local fracture risks or mechanical antiresorptive effects of implemented therapies [111,225].

Considering the limited incidence of MM and OS, individual centers rarely provide enough patient numbers for training robust in silico models. Federated learning (FL) frameworks trained locally at each institution may address the limitation by enabling multi-institutional modeling [226,227]. Applied to MM and OS, FL infrastructures may build and validate models that generalize across institutions, health systems, and demographic groups [228].

## 7. Materials and Methods

This study presents a structured narrative review aiming to provide the first integrated comparative analysis of MM and OS through a computational modeling lens. As a structured narrative review, it does not follow a formal systematic review framework. Additionally, it does not aim for exhaustive or unbiased literature coverage.

This review was carried out to summarize the recent literature on computational modeling of bone cancers, with a specific focus on the cross-pathology transferability of multiple myeloma and osteosarcoma. The authors primarily considered references from 2020 to 2026 to ensure the relevance of the findings. The research was conducted using Google Scholar, Web of Science, PubMed, Scopus, and other resources. The focus was on bone cancer, multiple myeloma, osteosarcoma, computational models, and artificial intelligence. Specific search terms, or a combination of the following keywords were used to find pertinent research articles: “bone microenvironment”, “bone cancer”, “bone tumor”, “oncology”, “multiple myeloma”, “osteosarcoma”, “computational”, “in silico”, “artificial intelligence”, “mechanistic model”, “machine learning”, “digital twin”, “virtual cohort”, “PBPK”, “MIDD”, “RANK/RANKL/OPG”, “Wnt signaling”, “PI3K/AKT/mTOR”, “cross-cancer comparison”.

For each study, the focus was on the effects of disease pathophysiology on the mechanism generation and its results as well as the way to translate it in computational approaches. The reviewed computational models were classified according to their structure (mechanistic, ML, and hybrid), role (digital-twin and virtual cohort), and pharmacology context (MIDD/PBPK), permitting both MM and OS frameworks to be associated with multiple tags, e.g., mechanistic-QSP and digital twin.

The criteria for inclusion and exclusion into this research were based on the applicability of the studies to the molecular characterization, disease mechanism, diagnostic classification, or therapeutic approach of MM and OS. Regarding tumor types, the inclusion criteria were limited to MM and OS. These two malignancies benefit from extensive multi-omics characterization and well-known dependence on the bone microenvironment [229,230,231]. Articles that employed in silico modeling, molecular profiling, computational bioinformatics, and AI-driven methods were prioritized, in addition to other methodologies tailored to these two cancers. Studies were considered eligible if they met the following criteria: (i) peer-reviewed original research articles, reviews, or methodological papers published in English; (ii) addressing the pathophysiology, molecular biology, genomics, or tumor microenvironment of MM or OS; (iii) describing, applying, or evaluating computational, mathematical, or in silico modeling approaches in the context of MM, OS, or bone malignancies.

Publications were excluded based on the following criteria: (i) if they were published before 2020; (ii) if they addressed other malignancies than MM or OS (for example, Ewing sarcoma or chondrosarcoma were excluded due to their multi-omics and integration deficit, limiting the development of robust and replicable in silico approaches. Additionally, they are defined by key molecular factors that describe more lineage-specific oncogenic pathways, such as EWSR1::FLI1 fusion oncoprotein in Ewing sarcoma [232] and IDH1/IDH2 mutations in chondrosarcoma [233]. These molecular features are not directly comparable with the pathway aberrations of MM and OS. In a similar way, other hematological cancers, including acute myeloid leukemia, chronic lymphocytic leukemia, or non-Hodgkin lymphoma, were excluded since they do not primarily involve bone destruction or remodeling, the main biological axis underlying this comparative study); and (iii) if they described computational models that were not linked to bone diseases.

Next, the included studies were divided into distinct themes based on the tumor type and computational method. An additional focus was on common biological features and computational strategies that can be transferred from one disease to another. Strengths and limitations of the reviewed studies were also addressed, although there is some imbalance in the depth of available knowledge between the two malignancies. This work employs a qualitative, conceptually driven synthesis organized by theme across the manuscript.

## 8. Conclusions

This review provides a critical comparison of links between computational models, human cancer research, and the clinical pathophysiology of a disease for multiple myeloma and osteosarcoma. They are two distinct malignancies that share common features, providing a unique opportunity to enable the transfer and integration of approaches across cancer domains. From this point, it can reinforce both joint analysis and disease-specific research, which, in turn, can advance the understanding of each oncologic entity. To the best of our knowledge, this review represents the first structured comparative approach to computational strategies, specifically designed to bridge MM and OS, developing a conceptual and methodological basis for future in silico cross-disease oncology research.

MM can be considered as having a well-defined mechanistic basis regarding bone niche dynamics or pathway signaling, while OS is more considered with AI- and radiomics-based designs. Cross-disease comparative modeling can allow OS studies to integrate the validated bone marrow and immune designs originating from MM. At the same time, MM research can benefit from ML and imaging-informed frameworks implemented for OS. Hybrid mechanistic–ML approaches and digital twins may also be used as a convergence point since both cancers share the same pathways, for example, RANK/RANKL/OPG or PI3K-AKT-mTOR, becoming reusable modular computational components. A key observation from this comparative study is the significant computational asymmetry between these two malignancies. MM is supported by a more mature in silico modeling infrastructure, while OS has limited digital-twin approaches and validated ML frameworks. This gap in knowledge may benefit from cross-disease knowledge transfer.

Artificial intelligence, such as LLMs or foundational models, represents a powerful tool, not to replace any of the computational models, but to enhance capabilities to promote a deep, multimodal, and real-time update of current knowledge and support the advances towards precise forecasting in bone cancers. Nevertheless, their incorporation into MM and OS clinical procedures is still a developing approach that needs prospective confirmation prior to clinical use.

Most of the cross-disease computational frameworks addressed above are still in the proof-of-the-concept or early developmental stages. Their clinical translation will necessitate interdisciplinary cooperation among computational sciences, oncologists, and regulatory agencies as well as prospective validation and standardized multi-omics datasets. The concept that a unified cross-disease in silico approach to MM and OS has the potential to speed up treatment development and improve precision oncology for both cancers is supported by these data taken together.

## Figures and Tables

**Table 1:** Common and individual key features of MM and OS linking biology with computational modeling.

Focus	Specific Features		Common Features	Framework	Limitations
	MM	OS			
Oncogenic network	del(17p)/TP53 loss, MAPK-MYC axis [115] PI3K-AKT-mTOR [116]	PI3K-AKT-mTOR, Wnt/β-catenin, Hedgehog, Notch [117] SOX2, NANOG, OCT4 [65]	Sustained proliferation, impeded apoptosis and immune resistance	Mechanistic/QSP Data-driven/ML	Over-simplified signaling [40] Lack of spatial detail [118] Immune evasion [119]
Bone niche	RANKL-osteoclastogenesis [120] High glutamate and RANKL via NF-kB-NFATc1 [121]	Osteoid and pre-metastatic niche activation [122] CXCL14-integrin-TGF-β, IL-1 [123]	Bone–immune–stromal signaling, bone cells remodeling	Mechanistic/QSP Hybrid/Mechanistic learning Digital twin	Low osteocyte-specific regulation and metabolic inputs [120] Simplified bone–lung correlation Lack of spatial detail [124]
Immune and cytokine milieu	TGF- β1 and IL-10 [125] CCL3-HMGB1-RANKL [120]	IL-1 β, IL-6, TNF, CXCL, TGF-β1 [126] M2-like TAM2 [127]	Immunosuppressive microenvironments Cytokine release	Hybrid/Mechanistic learning Digital twin/Virtual cohort PBPK/MIDD	Low integration of multiple cytokines [128] Rarely modeled multiple tumor-secretor factors [129]
Dataset	scRNA-seq [130] and MSC profiling [131]	scRNA-seq [132] and secretome identification [133]	MM with dense data landscape OS high-value multi-omics	Hybrid/Mechanistic learning Digital twin/Virtual cohort	MM with limited radiomics and spatial omics of bone lesions [134] Insufficient data for OS [135]

**Table 2:** Advantages, limitations and common features of computational models with implications for MM and OS.

Model	Advantages	Limitations	Common Features
Mechanistic	Interpretability; Ability to simulate drug schedules, combinations, and resistance mechanisms [60,87]	High model complexity; Poorly constrained parameters for bone biology and immune crosstalk; Lack of disease-specific calibration data [86]	Cellular signaling pathways [77,195]
Data-driven/machine learning (ML)	Enhanced prognostic and therapeutic outcome predictions; Multi-modal integration; Transferability features [173]	Potentially limited external validation of AI studies; Data imbalance: MM > OS [115]	Inter- and intra-tumoral heterogeneity across molecular, imaging and clinical features [196]
Hybrid/mechanistic learning	Potential for integration of AI/ML into patient-tailored strategies with continuous update [188]	High model complexity with dependence on datasets [197,198]	Mechanistic core with data-driven observation layer [137,199]
Digital-twin/virtual cohort	Provision of in silico virtual single-patient-specific or cohort trials for precision medicine [101,178]	Lack of standardized clinical workflows; Ethical and legal concerns; Data imbalance: MM > OS [200]	Common digital-twin backbone [97]
MIDD/PBPK	Support for trial frameworks and linking dosing, exposure and systemic toxicity [201,202]	Constraint for accurate representation of micro-scale bone architecture and dynamic reorganization [203,204]	Shared pharmacology backbone, AI-reinforced [205]

## Data Availability

No new data were created or analyzed in this study. Data sharing is not applicable to this article.
